# Preparation for Meaningful Work and Life: Urban High School Youth’s Reflections on Work-Based Learning 1 Year Post-Graduation

**DOI:** 10.3389/fpsyg.2016.00286

**Published:** 2016-02-26

**Authors:** Maureen E. Kenny, Christine Catraio, Janine Bempechat, Kelly Minor, Chad Olle, David L. Blustein, Joanne Seltzer

**Affiliations:** ^1^Department of Counseling, Developmental, and Educational Psychology, Lynch School of Education, Boston CollegeChestnut Hill, MA, USA; ^2^Counseling Center, University of New HampshireDurham, NH, USA; ^3^Wheelock CollegeBoston, MA, USA; ^4^University of TexasAustin, TX, USA; ^5^Curry CollegeMilton, MA, USA

**Keywords:** work-based learning, post-high school transition, school to work transition, college access, decent work, employability of low-income youth

## Abstract

The challenges confronted by low-income high school students throughout school and across the transition to higher education and employment are well-documented in the US and many other nations. Adopting a positive youth development perspective (Lerner et al., 2005), this study reports findings from interviews with 18 low-income, racially and ethnically diverse graduates of an urban Catholic high school in the US. The interviews were designed to shed light on the post-high school experiences of urban high school graduates and to understand how students construct meaning about the value of school and work-based learning (WBL) in their preparation for meaningful work and life. The interviews highlight the perceived value of the academic and non-cognitive preparation students experienced through high school and WBL in relation to the challenges they encountered along the pathway to post-high school success and decent work. Overall, the findings suggest the potential of WBL for low-income youth in facilitating access to resources that build academic and psychological/non-cognitive assets, while also illustrating the role of structural and contextual factors in shaping post-high school transitions and access to meaningful work and life opportunities.

## Introduction

Within the US, low-income youth of color, many of whom reside within and attend schools in large urban centers, have long experienced barriers in accessing decent work that offers a living wage and opportunities for advancement. In recent years, opportunities for stable employment have diminished, along with increases in underemployment and growing income disparities between the rich and the poor. Across the globe, 75% of workers are employed in temporary or short-term positions, contributing to the widening income inequality between those who are more aﬄuent and highly educated and those who are not (International Labour Organization [ILO], 2015). The International Labour Organization [ILO] (2008), an agency of the United Nations, maintains that all workers should have access to decent work that is integral to personal and family well-being and to social and economic advancement. According to the International Labour Organization [ILO] (2008, 2012), decent work should offer a fair income, protection from wage loss, safe working conditions, access to health care, respect for social and family values, and opportunities for social dialog and worker organization. Although the ILO purports that sufficient decent work opportunities should exist, such that everyone who wants to work has the opportunity, decent work is diminishing while temporary and part-time work are increasing (International Labour Organization [ILO], 2014).

As decent work opportunities in the US workforce shrink as a result of technological change and turbulence in the economy worldwide, young people from low-income backgrounds, who have less education, and who belong to a racial or ethnic minority group are often marginalized from the economic, academic, and social resources needed to compete for the stable and decent work opportunities that do exist (Casey, 2012). Indeed, while many young people across the globe are challenged in their efforts to secure decent work, those who are poor and from marginalized groups worldwide experience the greatest obstacles (Blustein et al., 2014; Quintini and Martin, 2014). This study focuses on elucidating the post-high school experiences of graduates of a US high school designed to prepare young people from urban and low-income families for successful futures through work-based learning (WBL) and entry into higher education and decent work. By asking high school graduates from low-income backgrounds to reflect on how their high school experiences prepared them for life and work, we hope to gain insights into how students construct meaning about school and work, and the perceived value of school and WBL in their preparation for the future. These insights might inform further research and efforts to enhance the life chances and access to decent work for those young people most disenfranchised due to economic and social barriers.

Education at the high school level and beyond is a precursor to attaining gainful employment and access to associated economic, health, and social benefits. Within the US and globally, persons with higher educational status are less likely to be unemployed and more likely to enjoy physical and psychological well-being across the life span (Fouad and Bynner, 2008; Kenny and Walsh-Blair, 2012; OECD, 2014). Technological change in the workforce and the elimination of many unskilled jobs due to automation and outsourcing means that advanced education is increasingly critical for entry and advancement in the world of work (International Labour Organization [ILO], 2015). Despite the importance of advanced education, 19% of US high school students do not graduate on time with a traditional high school diploma (National Center for Education Statistics [NCES], 2014) and 20% of those who do graduate, enter college needing remedial courses (National Center for Education Statistics [NCES], 2013). Academic under-attainment is most prevalent among young people growing up in a context of economic poverty, who often attend schools staffed with less experienced teachers and with high teacher turnover (Reardon, 2011). These students experience an “opportunity gap” in comparison with more aﬄuent students and are more likely to drop out of high school, reap less income over their lifetime, and belong to the ranks of the unemployed for longer periods of time (Cauthen and Fass, 2008; Reardon, 2011). Among high school graduates, lower-SES youth are also less likely to apply to and enroll in postsecondary education and are less likely to complete a 2 or 4-year degree than students from high-income families (Walpole, 2007).

In addition to academic and technical skills, a host of non-cognitive or soft skills, are recognized as important for success in finding and retaining a job, advancing at work, and attaining a higher income and greater work security (Lippman et al., 2015). Indeed, an individual’s employability, or capacity to obtain and retain suitable employment, entails not only vocational or job-related knowledge and skills, but an array of psycho-social skills that enable individuals to adapt to the changing demands of the world of work and to identify and realize work opportunities (Fugate et al., 2004; McArdle et al., 2007; Rothwell, 2015). A growing body of evidence across the fields of psychology, sociology, economics, and positive youth development (PYD) attests to the importance of this array of varied but interrelated non-academic skills for doing well in work and school settings, achieving desired goals, and relating effectively with others (Richardson et al., 2012; Lippman et al., 2015). This broad set of skills has been conceptualized and studied based on varying frameworks and rubrics such as developmental assets, life skills, character, grit, career adaptability, emotional intelligence, social-emotional learning and 21st century skills, among others (Farrington et al., 2012; Savickas and Porfeli, 2012; Kenny and Minor, 2015; Rothwell, 2015). While each of these frameworks is unique, some shared constructs emerge that relate to self-control, social and communication skills, self-awareness, self-confidence and positive attitudes for the future (Watts, 2006; Lippman et al., 2015; Rothwell, 2015). Despite the variety of skills and inconsistencies in defining and naming the constructs, a converging body of research suggests that these skills are malleable in the adolescent years and have an impact on academic success, engaged citizenship, PYD, career decision-making, workplace success, and life quality in general (Di Fabio and Kenny, 2011; Di Fabio and Maree, 2012; Richardson et al., 2012; Di Fabio and Saklofske, 2014; Lippman et al., 2015). Evidence is also emerging that these varied non-cognitive skills are especially important for sustaining career progress and maintaining personal well-being during times of economic and social uncertainty (Guilbert et al., 2015; Kenny and Minor, 2015; Di Fabio et al., in press). Moreover, employers in the US and other nations are expecting employees to be ready for the workplace equipped with non-cognitive assets as well as academic and vocational skills (Casner-Lotto and Barrington, 2006; Watts, 2006; Rothwell, 2015).

While we maintain that broad systemic change is important for reducing the range of structural inequities that create barriers to decent work, we also believe that high quality school and out-of-school experiences can help to equip young people with the academic and non-cognitive skills vital for establishing a trajectory of positive academic, social, and vocational development. The PYD perspective (Lerner et al., 2005), which is anchored in developmental systems theory (Lerner, 2006), emphasizes the important role of schools and other youth-serving settings in offering experiences and supports that enable youth to develop the individual assets and competencies needed to thrive throughout adolescence and into adulthood. According to developmental systems (Lerner, 2006) and PYD (Lerner et al., 2005) frameworks, positive development results from an alignment between contextual and individual assets. With regard to individual assets, PYD recognizes the importance of academic and non-cognitive skills for young people to thrive in school, work, and life (Lerner et al., 2005). Schools represent one vital context that can play an essential role in teaching academic skills and providing opportunities to develop the non-cognitive skills and social and emotional competencies that are vital for lifelong success (Watts, 2006; National Research Council, 2012).

Work-based learning, which encompasses learning in the workplace through internships, apprenticeships, informal learning on the job, and other vocational-specific curricula, has engaged interest in many countries around the globe for its potential to facilitate transitions from school to work, to ensure that training is aligned with labor market needs, to provide opportunities for the ongoing development of transferrable skills to meet the needs of the changing workplace, and to help students understand and adapt to the realities of the workplace (Falconer and Pettigrew, 2003; OECD, 2014; Hoffman, 2015). Although WBL programs are more often offered across higher education and vocational training, WBL has also demonstrated promise at the secondary school level as a model for connecting academic learning to work preparation, enhancing positive student attitudes, academic motivation and goal articulation for school and career, and equipping young people with both academic and non-cognitive work readiness skills for the transition from high school into meaningful work and life (Visher et al., 2004; Blustein, 2006; Kenny et al., 2010; Bempechat et al., 2014; Quintini and Martin, 2014). WBL programs thus represent one youth setting that can potentially complement the academic learning environment to develop personal, social, and work readiness competencies. Although some countries, such as Switzerland, Germany, Singapore, and the Netherlands, have highly developed or comprehensive WBL programs at the secondary school level, WBL programs are not typical or systemic in the US (Hoffman, 2015).

In response to the concern for improving the academic achievement of youth growing up in poverty, the Cristo Rey network, an association of 26 Catholic high schools across the US, emerged, with the intent to “transform urban America, one student at a time” (Sweas, 2014). The schools, which serve only youth from low-income families, integrate a number of unique features, with the intent to provide a holistic education that fosters academic, social, civic, moral, and spiritual development. In addition to offering a faith-based education, they provide an academically rigorous college preparatory curriculum coupled with WBL. In the Cristo Rey WBL model, students engage in real work that the corporation would otherwise pay another individual to do, with the salary paid by the corporation going toward the student’s high school tuition (Thielman, 2012). Typically one full-time entry-level position is split among four students from the school with each student working 1 day each week. Every student works across all 4 years of high school in a corporate or non-profit setting, with a focus on developing non-cognitive work readiness skills, rather than the development or transfer of vocational or technical skills to the workplace.

The present qualitative study is designed to enhance understanding of students’ perceived academic and non-cognitive skills, the perceived value of their high school experiences, and their experiences across the post-high school transition to higher education and work. Underlying this study is an ethical stance that builds on the Jesuit origins of the Cristo Rey Network (Kearney, 2008) and analogous movements in psychological studies of education and work, which collectively affirm the importance of establishing equality and social justice in the systems that frame students’ development. When considering the Cristo Rey ideology in conjunction with recent calls for justice in scholarship and program development efforts in career development (e.g., Psychology-of-Working framework; Blustein, 2006), the commitment to unpacking sources of marginalization and resources that help students counter these oppressive forces serves as a cohering aspect of our project. Among students who graduated from an urban Catholic high school affiliated with the Cristo Rey Network, we explored graduates’ perceptions of their adaptations to education, work, and life 1 year post-high school and the meanings they ascribed to their high school academic and WBL experiences in relation to their current lives. Students’ perceptions of their challenges and the value they assigned to their high school experiences may provide valuable insights on how low-income urban youth understand their personal pathways toward higher education and the adult world and the types of experiences they view as facilitating and impeding their progress. This study will help to advance knowledge about the potential of intentional WBL as a viable means of promoting access to decent work among a population of youth who are often marginalized from the resources needed to find meaning and purpose in their adult work lives.

## Materials and Methods

### Participants

A total of 18 low-income racially and ethnically diverse high school graduates (12 females; six males; eight Black, nine Latino, and one White) of Cristo Rey Northeast (a pseudonym) participated in this qualitative study. The WBL model at this school includes weekly one full-day per week in the workplace, a weekly holistic reflection seminar at the school during which students discuss their workplace experiences, and quarterly student evaluations by work site supervisors. The participants in this study had worked during high school in WBL positions in a variety of settings, including a biogenetics laboratory, a high school administration office, the computer lab at a major university, the corporate offices of a national retailer, a public aquarium, and a homeless shelter. Although many students enter this high school with academic skills significantly below grade level based on standardized tests, they often make substantial academic gains during high school, with most graduates being accepted to 2 or 4-year college programs (Thielman, 2012). Although the 18 participants in this study are a volunteer sample of high school graduates, they achieved varied levels of academic success in high school. The average cumulative high school academic grades for these graduates, obtained from school records, ranged from 1.22 to 3.98 on a four-point scale (with 4.0 reflecting an A), with a mean of 2.37, which is very close to the 2.41 mean for all students from the same graduating class.

### Procedure

All 60 members of the prior graduating class were invited by mail to an alumni event held at the high school on graduation day, 1 year after their graduation. The research team spoke to the approximately 40 alumni in attendance, explained the research study, and invited graduates to participate in interviews about their post-high school experiences. Those willing to share their experiences were individually interviewed for approximately 30–45 min. The procedure was approved by the high school administration and by the university committee that reviews and approves faculty research to ensure the safe and ethical treatment of research participants.

### Interview and Analysis

The interview consisted of open-ended questions that tapped students’ perceptions of their transition from high school to college/vocational program/work, as well as their reflections on their high school and WBL experiences. The complete interview is provided in the Appendix (Supplementary Material). Interviews were taped and transcribed verbatim, and subsequently coded by the research team using thematic coding and content analysis (Bempechat et al., 2013), which is described more fully in the following paragraphs.

Research team members read the first interview individually to identify emerging categories and then met as a group to compare initial codes. This process was repeated with other interviews and distinct categories were identified through the use of constant comparison (comparing the text of interviews in a category for similarities and differences; Glaser and Strauss, 1967), resulting in a codebook with category definitions and examples. Coding was both deductive, using the structure of the interview questions and existing theory to identify meaningful themes, and also inductive, with the research team members looking for nuanced and context specific themes that may not have been directly elicited by the structure of the interview or highlighted in existing literature. As new categories emerged, the research team re-coded the interviews as part of an iterative process, resulting in a total of 23 categories. The initial 23 codes are closely aligned with the interview questions and include student activities in the first-year post graduation; their major responsibilities across the past year; their expectations prior to graduation; their actual experiences in the past year with subcodes for difficulties, surprises, and other; their perceived preparation from their high school, with subcodes for the educational program, religious orientation and WBL work site experience and holistic reflections seminar at the school; their perceptions of social support across the post-high school year; student perceived growth in post-high school year; comparison of their maturity to peers; their future goals, among other codes. (A copy of the complete coding booklet is available from the authors by request).

The 23 categories were reviewed and grouped individually and then collectively by the research team into four broad themes: students’ actual experiences post-graduation, their future goals, their perceived challenges and assets, and their perceived academic and personal preparation from their high school and WBL experiences. The categories and content organized under each theme was synthesized into four memos or summaries. The research team members wrote, reviewed, and discussed the summaries and met to further clarify, refine, and elaborate on the overarching themes and to determine descriptive labels for these themes and subthemes. For example, the memos and the codes aligned with each memo were reviewed individually and collectively by members of the research team, who identified perceived post-high school challenges, the varied and specific personal qualities that students felt that they had developed during high school that helped them to navigate these challenges, and their attribution of these personal qualities to the academic, WBL experience, or holistic-reflection components of their high school program as overarching themes that, in addition to their stated post-high school activities and future goals, spanned the four memos. The consensus on the overarching themes apparent across all interviews was reached through a process of review and discussion among the team members.

The interviewers, transcribers, and coders included three university faculty and graduate- research assistants from three collaborating colleges and universities in the metropolitan area surrounding the participants’ high school. Members of the core research team identified as four White women and four White men, with two Asian–American women, one Latina woman, and one Black woman also involved in various phases of this study. The researchers continually addressed possible biases and evaluated inter-rater agreement by having two-person teams code the same interview individually, and then meet to compare codes. In addition, the team members were cognizant of the ethical stance that underscored their work on the project, which helped to ensure that the interpretation of the findings incorporated a clear vision of how social and economic forces shape students’ experience. Furthermore, a knowledgable auditor was present at the weekly meetings to review interviews, facilitate critical discussion, challenge assumptions, pose alternative interpretations, ensure all opinions are considered and facilitate consensus (Morrow, 2005). When considered collectively, these procedures are designed to contribute to the validity of the results.

## Results

### Overview

The interviews revealed that the alumni had engaged in a variety of school and work activities since their graduation. Although all of the graduates had been accepted into some form of higher education, not all graduates actually enrolled and some left before completing their first year. The post-high school transition was relatively smooth for some, while others encountered life events that altered their immediate plans.

Across the 18 interviews, several broad themes emerged, which describe the ways in which students viewed their development and preparation for the future as emerging through their unique high school experiences and their experiences in the transition from high school to work and/or higher education. Although not all graduates were enrolled in higher education at the time of the interviews, all described a sustained focus on pursuing further education and felt that their high school had helped prepare them in important ways for post-high school life. That is, in addition to the academic preparation provided by their high school, all students described personal qualities developed over the course of high school that prepared them for school and work post-high school. The narratives reveal furthermore how students perceived those qualities as having been fostered through the unique education and WBL settings in which they were immersed.

### Post-High School Experience

The fall following high school graduation, 11 of the 18 alumni enrolled in college programs varying in level of selectivity. Of the 11 college students, two were also working full-time to support themselves. One was working full-time as a collections officer and the other was working in retail sales in a shoe store. Two left college after one semester. One left to due to the medical complications of a pregnancy and financial concerns, but planned to enroll in a community college the next fall, with another leaving for financial reasons. The latter was a very strong student academically in high school who had made a successful academic adjustment to a 4-year college while working part-time in a real estate office. She left after the fall semester, obtained her real estate license and was then working full-time in real estate sales with plans to return to college in the fall after saving some money. Three graduates were enrolled full-time in vocational or technical preparation programs for training as a massage therapist, a pharmacy technician, and an electrician. Four graduates had delayed their plans for post-high school study and were working at the time of the interview, yet expressed a firm commitment and evidence of active planning to enter college. One of the graduates delayed attending college in order to care for her mother who became ill and to look after a younger sister. She was working full-time as a sales supervisor in a small department store. Another delayed college for a year because she was undecided on a course of study. She was working at a jewelry counter in a department store, with plans to attend college in the fall. Another graduate delayed his plans to enroll in college after her parents decided to return to their county of origin. This graduate was now working in food services and living with siblings who remained in the US, with plans to attend community college in the fall. The final graduate, who was working full-time as a waitress and at the counter in a restaurant, had always planned to attend college, but did not think she could afford to go at this time. A summary of graduates and their post-high school experiences is provided in **Table 1**.

**Table 1:** Summary of graduates and their post-high school (post-HS) experiences.

Post-HS experiences	Student	Gender	Race/Ethnicity
Direct to 4-year college			
1	Vanessa	Female	Black
2	Marcos	Male	Latino
3	Alberto	Male	Latino
4	José	Male	Latino
5	Karen	Female	Black
6	Linda	Female	Black
7	Jessica	Female	Black
Direct to 4-year college part-time (simultaneously with full-time employment)			
1	Sebastian	Male	Latino
2	Leyla	Female	Latina
Direct to 4-year college (interruption in enrollment)			
1	Maya	Female	Latina
2	Judy	Female	Black
Direct to community college, vocational, or technical school			
1	Cassandra	Female	Latina
2	John	Male	White
3	Roman	Male	Latino
Direct to full-time employment (no higher education)			
1	LaToya	Female	Black
2	Janet	Female	Black
3	Monica	Female	Latina
4	Maria	Female	Latina

### Future Orientation

Although not all of the graduates were enrolled in post-high school study 1year following graduation, all described a future orientation that included continuing their education. Twelve of the 18 alumni described a clear career goal that was grounded in a direct route from high school through college or vocational school. The remainder expressed more uncertainty or the presence of life events that altered the clarity of their path.

Karen (all names are pseudonyms), studying health science at a 4-year college, exemplified those alumni expressing a clear career focus and pathway toward her goals. She stated:

Right now my goals are pretty simple. My goal for school is to keep going at the rate I’m going. I want to get my GPA (academic grades) up. After that I want to get to med school by the end of the 3 years that I have left. My goal for summer is just to get a job, and get a permit for my car.

Roman described a clear sense of direction through vocational training:

It’s like a 9-month program just to get the apprentice license and then after that I have to join a company for 4 years, get my hours to go to my journeyman’s license, then one more year to get my master’s license and then I can be a master electrician, own my own business.

Vanessa, attending a 4-year college and studying biochemistry, felt that her career direction was stronger than other students at her college. She explained:

It’s really strange to me to see the people even in my science classes right now telling me that they want to go to med school, but not exactly knowing how to get there… I think that internship here, the work study fills in that gap.

Although all of the alumni were not as clear about their career direction, they nevertheless expressed a firm commitment to post-high school study and future planning.

Maria, who was working full-time in food services to save money, described her strategy for getting to college:

Yeah, I am going to get another job because I want to save as much money as I can to pay for college, because I always felt that when I went to college I wanted to have a part-time job, and I would just be studying and study hard and that’s what I really want to do.

Jessica, who was attending college full-time, discovered that she no longer wants to be a nurse, but expressed a commitment to continuing in college and identifying a new career goal:

I went in as a nursing major, but quickly found out that’s not what I want to do. So, I continued as an undergrad student, and now I’m taking summer school to kind of find my niche as to what I really want to pursue as a career.

In considering possible options for the future, Jessica reflects on her WBL experience at an aquarium during high school: “I liked to do … work there [at the aquarium], so I could have that type of experience when I try to pursue my career as a veterinarian…”

### Alumni’s Perceptions of Challenges and Preparedness

The alumni noted varied challenges in their post-high school year as well as ways in which their high school and the work-study programs helped to prepare them for life after high school. With regard to challenges, alumni commonly mentioned the need to hold themselves responsible with less adult supervision and the amount of work required in higher education. Judy, who left college to have a baby and planned to return the following fall, described the challenge of adult responsibilities. She explained:

A lot of work to live life after high school. Like I said before, you know, you have to grow up. You’re an adult now you’re not just a student. You have to try to, like some people have to live on campus and try to make it on their own, you don’t have your mother there at all times.

Leyla, who was working full-time and attending college part-time, commented, “I mean it really isn’t easy. You get a lot of free will, you’re not forced to go to class, you’re not forced to be there, but you need to know the consequences if you’re not.” In comparing the demands of her life in high school and college, she noted that she is “working every day of the week, not just 1 day. So it’s a lot harder.”

Among those who enrolled in higher education, all students felt prepared academically, although several identified the increased workload and higher expectations as challenging. With regard to academic preparation for college, Vanessa stated:

Northeast prepared me really well for it…I give most of my credit to …the English department [at Northeast]. Science was not tough at all like I thought it was going to be…It was all very much like stuff I had learned here.

Roman found the academic work at the technical school easier than in high school, so he enrolled in community college to take more advanced math coursework in pre-calculus. Maya, who left college for financial reasons after one semester, found the academic work manageable: “I thought in my mind that it would be a lot harder…but I guess I was well-prepared and it got me in a good position in college. For me, it wasn’t hard at all.”

Linda, a college pre-med student, felt prepared academically, but was among those who commented on the work volume. She noted:

I was prepared because of the way Northeast prepared us. Um, we not only went about going to school, but we also, basically had jobs while we were here. So as far as me being prepared to work, I felt like that, I felt like that I was. The only thing I wasn’t prepared for was the amount of work, because obviously in high school we don’t get as much work, and as time-consuming work as you would call it.

In addition to academic preparation, alumni highlighted their preparedness for post-high school life as encompassing three sets of interrelated non-cognitive skills, including a sense of self-control and self-regulation, self and other awareness, and social skills and professionalism. Although all of the alumni articulated ways in which their high school experience had prepared them for the year following graduation, they highlighted different benefits.

### Self-control and Self-regulation

More than half of the graduates spoke about their preparedness in relation to qualities of self-control and self-regulation. These qualities were viewed as important with regard to persistence, time management, and relational management in their current post-high school, work, and education.

Alberto, enrolled in a 4-year college, was one of the graduates who spoke about the importance of hard work and persistence, stating, “Nothing comes easy. You have to work for everything you want. In college, in school, in anything. Anything you want, you have to work for.” Cassandra, who was enrolled in a massage therapy program, had struggled academically during high school, but described how she persisted toward completing her degree:

I wasn’t like the best, the best of grades and everything, but I hung on by a thread. And you know, I got to the point where my thread snapped and I wasn’t able to graduate with my class, but I did not let that stop me… . So, I came back here and did what I had to do and passed the work and I just continued on with my mission.

Marcos, a 4-year college student, was among the alumni who described the WBL program as instrumental in fostering his maturity. He stated:

And then I just look at the factors to my level of maturity and, I see, like any experiences in life and then I see my high school and the [WBL] program. That really helped my level of maturity. And then I just think, some of these students probably didn’t go to a corporate work-study program. The odds are that a lot of them probably haven’t and that could be a reason why their level of maturity is different from my level of maturity.

[… Northeast] helps students…learn so many things. You learn responsibilities. You learn time management. You learn all these things that can make a young adult a very good adult.

Self-regulation was also exemplified in social relations and choice of friends. Sebastian, who was studying computer science, commented, “I try and find people who are the same as me. You know, outgoing, but somewhat compatible to me. I don’t hang around people who smoke and drink, and what-not. And if they do, I just leave.” He noted how the work experience exposed graduates to a set of rules and consequences in the world outside of school:

Here [at Northeast], you’re like we’re following rules and [if] you messed up, this is what happens and, you know, it’s a guideline. When you step out of here you have to make your own guideline and you have to follow your own rules and you know the consequences of work, it’s not just detention anymore. But I like it. I feel I was well prepared.

Some graduates also noted, how the WBL experience had prepared them to manage multiple responsibilities. Maya, who left college after one semester to work full-time and save for her return to college, stated:

Having an outside job and going to school and having all other responsibilities helped me manage my life so much. And that taught me so much, like when I moved out of my parents’ house, I was prepared already because I knew that it’s not easy working and going to school, doing all that and paying bills. So I had an idea already.

Maya also described how the WBL program played a role in fostering personal strength: “The training that they gave us in the beginning (to prepare for the WBL experience), that definitely helped me so much. It gave me more character, personality, and I became, like, a stronger person.”

### Self-reflection and Other Awareness

The majority of the graduates described themselves as possessing a level of self and other awareness, which differentiated them from their college and work peers. As they described their self-awareness, 14 of the 18 graduates made frequent reference to their high school WBL experience, and to the holistic reflection seminar that was designed by the school to accompany the WBL program.

Roman, in technical school training to become an electrician, described the WBL program as fostering awareness of self and the world beyond high school:

I mean, a lot of the kids here were just kinda closed off to what the real world was and then…now with the internship program, it kinda opens up, you know, everyone’s minds, how it actually is out there, and I kinda like seeing that.

Monica, who took a year off to earn money to pay for college, also viewed the WBL experience as fostering awareness of the larger world. She stated:

But if you work outside the school, you’re working with new people with different attitudes, different people that don’t know you. And they’re starting to know you each day…each day that you go there … You see what I mean? It’s like stressful, it’s really stressful. But if you work in a different place, your mind’s gonna think differently, you’re gonna do things differently, you’re gonna be with different people.

Other graduates commented on how the weekly holistic reflection seminar held at the school taught them how to be self-reflective and learn from past experiences, thereby enhancing self-awareness and self-understanding. For example, Sebastian, studying computer technology at a 4-year college, commented, “…when we did the reflections about work and stuff. It helped me a lot to think about the way I deal with certain situations at work now.”

Liz, who was working full-time in retail, also spoke of the benefits of the holistic reflection seminar:

It was just good to talk about work and just reflect on everything that happens. And sometimes I needed that if I had a long week at work. Sometimes I just need to sit back and think about… oh maybe I should have done this, or maybe I should have done that. But, it did, it was pretty good. It did help somewhat.

Karen, studying health science at a 4-year college, also valued the lessons associated with the reflection seminar. She commented:

They had a lot of quotes, and a lot of things that told you to step back from the situation and look at it or like, you know, focus. Things like that help you realize that when things get really hectic, step back, look at what’s really going. And then deal with it. Kinda like small steps, is what I feel I took out of reflection.

Jessica, attending a 4-year college and undecided about her major, commented:

I love the holistic reflections. Um, they helped a lot, um, they’re very strategic in the way that they made the sessions … the questions that they asked us were very, very good. They help you think basically and helped you figure out ways to go about situations concerning school or the work study, how to go about it in a better way, a more constructive way, a more respectful way, and stuff like that. So it helped you to be more mature basically, it kind of helped me that way.

Cassandra, training as a massage therapist, appreciated the opportunity through the reflection seminar to express herself and get feedback from students and teachers. She said:

Being able to speak up, being able to be heard, being able to have people comment on what your issue is, being able to have somebody to relate to you who’s been there before, so you don’t feel like you’re alone…that helped me a lot too.

She also valued the personal and social knowledge she had learned from her teachers and the service experiences at the school:

There are two teachers here that I can say truly taught me the meaning of peace and community…there’s things I never knew I would do, like volunteer and get into the community, and just for them to put me at a homeless shelter. I was like, now I see things differently.

Cassandra developed a personal philosophy that helped her to persist when challenged. She explained:

There’s lessons in life that you got to learn…and you got to do something different. You gotta approach things differently. When you are in a situation, you look at both sides, put yourself in that person’s shoes…It’s certain things I ask myself just to get through.

Monica, who was working full-time with the intention of enrolling in community college in the fall, described her experiences in the reflection seminar and in interactions with teachers within the seminar and beyond:

They teach you to be proud of who you are, to become better at what you’re doing and what you do, to become good and feel prepared. They don’t want you to be ashamed…if you’re Latino… They want you to be a human being who makes a mistake and you’re able to learn.

Monica also attributed a heightened sense of self-awareness and confidence to her religion teacher, explaining:

She’s a really good teacher… she was always pushing me, like, “Roll through, just don’t get scared.” Maybe she’s more of a model for my life because she’s so self-confident. She’s not scared what people are thinking about her. Like she’s really strong religiously as a person. So, I learned that… that you need to be strong and fight for what you want and…that’s in my mind most of the time. So I really appreciate what she did for me.

### Social Skills and Workplace Communication

Many of the graduates described confidence in their social skills and communication for the workplace. Vanessa, a 4-year college biochemistry major, explained, “…I think like you’re always professional even though you don’t try to be. It’s just there. It’s just there, and I think they kind of ingrained that a lot in school…”

Relatedly, Cassandra commented on how she learned to communicate professionally through WBL. She reported:

…being able to be out there in the corporate world, being able to have, like, corporate communications, knowing how to be professional and knowing when to tone down your attitude. You know, once you step in the door, you know what I’m saying, like I said, it’s not about you anymore. It’s about who’s the client for the day.

The majority of the graduates commented explicitly on how the WBL experience had equipped them with valuable skills related to workplace communication. Jessica, enrolled at a 4-year college, gained an understanding of the value of a substantive resume through the WBL program. She stated:

I just want to express that the [WBL] program did help me a lot, um, I just filled out my resume, and when I was looking back at it, and really thinking about all the firms that I have been to, it’s just really a blessing to have experienced that.

Several students noted how the experience prepared them to interact with adults. Roman, studying to become an electrician, commented:

Like socially, it just helped me talk to adults, because I was so used to talking with people my age, and my boss was like fifty and I had no idea how to really approach her…but after the 2 years, we really became close and she was almost like a second mother to me because she gave me a lot of advice.

Marcos, a 4-year college student, reflected similarly:

…working at a corporate work-study program… helped me learn how to speak and how to communicate with adults … I need to know how to speak to an adult, how to like, say things in a way that an adult would understand. I can’t just use, I can’t just speak to adults the way I speak to my friends.

Graduates noted the importance of the social skills they learned while in high school for post-high school employment. Liz, who was working full-time in retail, while also caring for her ill mother, commented:

That is what [this school] is for, to prepare you for the future…Yeah, the working, and the dress code and how we all have to act professional and it is just a good set up for what is supposed to come after high school.

LaToya, who was working full-time with plans to enroll in college in the upcoming fall, also commented on the preparation she had received for the workplace, “I knew what to do and what not to do and being here they showed you how to dress for the corporate stuff, for the workplace.”

## Discussion

During the year after their high school graduation, the 18 young people interviewed in this study experienced varying levels of school and work successes and disappointments. They encountered many of the developmental challenges typical of young people their age, along with additional challenges, more common among young people who have limited access to economic resources and social capital as a result of their social status. While the transitions to higher education and work were relatively smooth for some of the high school graduates, for others the transition was marked by postponements and altered plans following unexpected life events. All of the alumni, however, maintained focus on further education and described a range of academic and non-cognitive skills that have been associated with workforce preparation and positive developmental trajectories (Watts, 2006; Lippman et al., 2015; Rothwell, 2015).

While growing up in low-income neighborhoods has been shown to have a profoundly deleterious effect on high school graduation rates (Wodtke et al., 2011), the young people in our sample exemplified some level of success through their completion of high school, acceptance into post-high school education, and entry into either higher education, employment or both. While statistics indicate that 15% of high school graduates in the US and 23% of those with less than a high school diploma between the ages of 16 and 24 are neither in school or employed (Bureau of Labor Statistics, 2014), all of the alumni we interviewed were engaged in school, work, or both. The percentage of young people who are neither in school or work is as high as 50% in countries with emerging economies (Quintini and Martin, 2014). In contrast to the prevailing criticism of high schools as not preparing young people for college and career (e.g., National Center for Public Policy and Higher Education and Southern Regional Education Board, 2010), these young people described their high school and WBL experiences as equipping them with varied academic and non-cognitive skills that they valued in navigating post-high school challenges.

The non-cognitive skills described by these young people are similar to those identified as valuable in prior research. Self-control, intentional self-regulation, and future orientation, for example, are highlighted in PYD research (Lerner et al., 2005). They are also similar to those identified by vocational psychologists as related to employability (Fugate et al., 2004; Rothwell, 2015), career adaptability (Savickas and Porfeli, 2012), and as critical for navigating life and work in a time of uncertainty (Di Fabio, 2014; Di Fabio and Kenny, 2015; Kenny and Minor, 2015). Conceptually related but distinct concepts, such as grit and perseverance have also received significant attention in recent research on youth academic and life success (Duckworth and Gross, 2014). In a broad sense, our alumni fit the descriptions of “gritty” youth, who demonstrate the capacity to regulate their time, attention, emotions, and social interactions, and sustain perseverance, despite ongoing life challenges. In addition to the intrapersonal skills associated with self-control and self-awareness, many of these young people described confidence in their social and interpersonal competencies, as well as capacities for self-reflection and self and other awareness, which have been identified as important competencies in research on social and emotional learning and for career decision making (Watts, 2006; Durlak et al., 2011; Di Fabio et al., 2013; Di Fabio and Saklofske, 2014; Rothwell, 2015). In this sense, these young people appear to be equipped with a number of the 21st century skills, which many employers observe as lacking in young people today (Casner-Lotto and Barrington, 2006).

Given the paucity of knowledge concerning the contextual bases for the development of non-cognitive skills (Farrington et al., 2012; Brown et al., 2013; Rothwell, 2015), the narratives offer insight into how some young people perceive their schools and specific aspects of their school programs as preparing them for life after high school. While the level and source of their non-cognitive skills are not verifiable from our data, these young people directly attributed many of these skills to the programs and relationships experienced in their high school. The idea that positive development for these young people stems from an alignment between their strengths and the assets of their context is consistent with the developmental systems perspective and PYD model (Lerner et al., 2005). The narratives of these youth reflect an understanding of human development as a dynamic interactive process in which the environment and the individual are both key in shaping outcomes. Recent attention to the role of “grit” (Duckworth et al., 2007) or perseverance toward long-term goals has been challenged by some critics, who maintain that that a focus on individual qualities may overlook the role of structural and systemic barriers that lie at the roots of the “opportunity gap” (Riele, 2010). Our findings serve to contextualize the understanding of non-cognitive skills, as alumni describe how real-world learning experiences in the workplace, the presence of caring adults at school and work (as well as in their families), and a space in the school curriculum to reflect on and give meaning to their work experiences contributed to their perceived non-cognitive strengths and preparation for the future.

Also consistent with developmental systems and PYD frameworks (Lerner et al., 2005), our findings reveal how contextual factors beyond the individuals and their school present substantive challenges for entry into and success in higher education and decent work. Youth with low economic resources often experience unique and complex hurdles in the transition to college (Rowan-Kenyon, 2007). Some of the graduates in our sample, for example, delayed post-high school study or dropped out of college for reasons less typical of the middle class college student, such as the need to support oneself when parents return to their country of origin or due to the complications of an unplanned and medically difficult pregnancy. Postponing college entry to care for a sick mother and sister was also cited as a reason for delaying college, perhaps associated with low economic resources and/or cultural values emphasizing family commitment and gender-based valuing of personal care work over market based employment (Richardson, 2012). Lack of economic resources, along with uncertainty regarding major or course of study, were also noted as reasons for not entering college immediately after high school. The life stories of the Northeast alumni are thus consistent with prior research revealing that low-income high school graduates may change their plans to enroll in post-secondary education over the summer months as a result of complicating life events and concerns about the costs and the economic benefits of higher education (Goldrick-Rab and Han, 2011). While the experiences of these students are uniquely embedded in the US context, they are consistent with global evidence documenting how economic and social barriers impede the academic and work achievements of young people growing up in low resource settings (Quintini and Martin, 2014).

The findings have relevance to exploring the challenges that face many youth as they transition from school to the world of work, and then optimally to an adult life that includes decent work. Their stories illustrate the inevitable interrelation of work and non-work experiences (Blustein, 2006) and the ways in which social, cultural and economic factors shape the journey from school to decent work (Guilbert et al., 2015). As reflected in the ILO’s contributions (International Labour Organization [ILO], 2008), many of the antecedents of decent work occur at the macro level, involving access to education, safe neighborhoods, good health care, and a host of other economic and social factors. However, individual and more proximal systemic factors also play a major role in the complex array of factors that promote decent work. As the narratives suggest in this study, the graduates of the WBL program at Northeast Cristo Rey faced numerous challenges once they left high school. However, some managed to make adaptive transitions to post-secondary training, while others struggled. The prevalence of external barriers seemed to overwhelm some of the participants, which underscores the need for equitable systemic solutions that will allow people to thrive and flourish in their educational and vocational lives. For many of the participants, though, the WBL experience was key in exploring the meaning of work in relation to their own values and to the development of a future orientation and an array of non-cognitive skills, which offered important internal resources in the struggle for meaningful work and life.

Although the alumni interviewed in this study highlight the capacity of schools and WBL programs to foster the development of non-cognitive skills in addition to academic development, the limitations of the study must be considered. With regard to generalizability, the participants represent a select sample of the high school graduates who chose to attend an alumni event. Although our participants experienced various levels of academic success during and after high school, it is likely that those students who felt disconnected from the high school did not attend the alumni event. The generalizability of our findings is also limited by the nature and structure of this particular school. This is a private, Catholic school in the US Northeast that serves only low-income students. The specific qualities of the students who choose to attend this school and the emotional support they receive from their families to enroll in and persist at this challenging high school are not clear. Although the findings from this study may be generalizable to other Cristo Rey high schools in the US offering a similar WBL model, the applicability for students in other WBL programs in the US and in other nations is not clear. The retrospective nature of students’ comments is, of course, subject to revisionist bias, and should be viewed with caution. The interviews were conducted only 1 year after graduation, so that the long-range post-high school success and career attainment of the graduates is unknown.

Further research is needed to address limitations of the current study. Follow-up of a random sample of graduates over a longer period of time is necessary to provide an understanding of their immediate and long-range experiences along a pathway to decent work. Future research should include the perspectives of key stakeholders from the high school, higher education, post-high school employment, and family contexts. Including individuals within these overlapping spheres of influence (Reschly and Christenson, 2009) can overcome the limitations of reliance on individual self-report and inform a broader understanding of how contextual and individual factors interact in creating and limiting access to decent work. Although, we found the PYD framework to be helpful in guiding this study, further research can serve to deepen understanding of role of socio-cultural, contextual, and structural factors in preparation for meaningful work and life and access to decent work for low-income youth. Varied stakeholders might further illuminate the internal, proximal and distal resources that young people might access in gaining entry into decent work. Further research at the distal level is needed to understand how workplace and broader economic policies interact with individual skills in impacting access to decent work. Comparing the experiences of graduates of this high school to graduates of other schools in the network across the US and other secondary school WBL models in the US and across the globe will also be important in gaining a broader understanding how students understand their high school experiences as preparing them for the future of decent work.

Given the limitations of this study, implications for policy and practice are considered tentative. In the context of ongoing educational policy debates in which creating a culture of college and career readiness is a priority in the US (Office of the White House Press Secretary, 2010), our study participants were able to reflect thoughtfully on how their high school and WBL experiences contributed to academic and non-cognitive skills that they have relied on to navigate myriad subsequent challenges. While we cannot infer causality and more research is needed, positive features of the WBL program and supportive and reflective learning experiences at school may be applicable in other school settings as vehicles to prepare low-income youth academically, socially, and motivationally for the ongoing challenges of young adult life. These characteristics have been noted in previous reviews of high school reform efforts in the US that highlight the importance of addressing the social, emotional, and motivational capacities youth, as well as their academic skills (Kenny, 2013) and have been noted in international research on effective WBL programs (OECD, 2014; Hoffman, 2015).

Beyond the high school and WBL contexts, our overall findings suggest the need for a multifaceted and systemic approach for enhancing access to decent work for low-income youth. Employability depends not only on well-developed individual skills, but also on organizational and broader contextual factors, such as the status of the labor market (Guilbert et al., 2015; Rothwell, 2015). Blustein et al. (2014) have labeled interventions that seek to enhance non-cognitive skills as promising micro-level initiatives potentially relevant in removing barriers to upward mobility, but acknowledge the necessity for further macro-level initiatives for work preparation, poverty reduction, and the expansion of decent work. The types of work in which most of the alumni were engaged reflect retail and food services positions that offer little long term security or room for advancement. Consistent with the PYD perspective, efforts to enhance resources across school, work, and public policy contexts are needed. The type of public-private partnerships exemplified by WBL might, for example, be expanded in creating more effective decent work pathways and opportunities for all young people (Blustein et al., 2014). WBL programs might also be enhanced, consistent with best practice in countries such as Switzerland and Germany, to provide a direct pathway from internships or apprenticeships to meaningful employment and to offer a system of ongoing career guidance beyond secondary school (OECD, 2014; Hoffman, 2015). Although recent US national initiatives suggest that all students who graduate from high school should be prepared for college and career (Office of the White House Press Secretary, 2010), our analysis suggests that some graduates may not have the interest or vocational clarity to pursue higher education immediately after graduation, such that enhanced and ongoing career counseling and post-high school employment might make sense for those figuring out a goal or course of study.

Although further research is needed to address limitations of the current study and to more fully understand the role of contextual influences, our findings give voice to the meaning that urban high school students ascribe to their high school and post-high school experiences, and offer insights concerning their varied pathways from high school toward higher education and meaningful work and life.

## Author Contributions

MK, CC, JB, DB, and JS were involved in the original review of the literature, design of the study, data collection and analysis, interpretation of the findings, and writing of the manuscript. KM and CO were involved in data analysis, interpretation, literature review, and writing of results and discussion.

## Conflict of Interest Statement

The authors declare that the research was conducted in the absence of any commercial or financial relationships that could be construed as a potential conflict of interest.
