# Supplementary material for: Preparation for Meaningful Work and Life: Urban High School Youth’s Reflections on Work-Based Learning 1 Year Post-Graduation
Source: Front Psychol. 2016 Feb 26;7:286. doi: 10.3389/fpsyg.2016.00286 (PMC4767925; doi:10.3389/fpsyg.2016.00286)
Supplement: Supplementary file 1 [file Data_Sheet_1.DOCX]

Appendix: Interview Protocol

*Thanks so much for agreeing to sit down and talk with me. We are studying the Cristo Rey work/study program, and it is especially interesting for us to talk with those of you who graduated last year. I’m interested in what you are doing now, how you experienced the transition from high school, and what your plans are for next year and beyond.* I. I’d like to begin by talking about your experiences this past year, since you graduated.

1. What did you do this year? [*Probe for details, i.e., if in college, where, full-time or part-time, course of study. If working or in apprenticeship, vocational training, probe similarly for details*]

2. Tell me about the transition from high school to college (or vocational school, apprenticeship program, etc).

3. How prepared do you feel you were for the academic challenges that awaited you? [Probe for work and school challenges.]

II. I’d like to continue by asking you to reflect on your experiences at Northeast High School.

4. Do you think the WBL program helped to prepare you for [college, work, etc]? If yes, in what ways? If no, why do you think not?

5. Are there ways in which your educational experiences at Northeast helped you for the next step? If yes, how? If no, how not?

6. What have you learned about life beyond high school?

7. Are there ways in which your experiences in the holistic reflection seminar helped prepare for the next step? Please explain.

8. Would you recommend to others that they attend a high school with a WBL program, like the one you experienced at Northeast? Why or why not?

III. Now I’d like to ask you about your current life.

9. Thinking about this past year (whether in college, working, military, etc.), can you please describe to me a typical day/weekend?

10. Thinking about the people your age you have met this past year, how do you feel you compare with them, with respect to preparedness or maturity? [*Probe for the individual’s perceptions*].

11. How is your current situation—where you are now in your life—similar to or different from what you expected a year ago? Please explain.

12. What are your current goals (personal, school or work-related)?

13. Do you have any other thoughts you would like to share with the research team?
